# Pembrolizumab in PD-L1-positive advanced non-small cell lung carcinoma: A meta-analysis of survival benefits and immune-related toxicity events patterns

**DOI:** 10.5599/admet.2956

**Published:** 2025-10-14

**Authors:** Alendra Chakramurty, Adetya Rahma Dinni, Ihda Silvia, Aprilyan Laras Cantika, Citrawati Dyah Kencono Wungu

**Affiliations:** 1 Department of Anatomical Pathology, Faculty of Medicine, Universitas Swadaya Gunung Jati, Cirebon, Indonesia; 2 Department of Pulmonology, Faculty of Medicine, Universitas Swadaya Gunung Jati, Cirebon, Indonesia; 3 Department of Internal Medicine, Gunung Jati General Hospital, Cirebon, Indonesia; 4 Department of Medical Education and Bioethics, Faculty of Medicine, Universitas Swadaya Gunung Jati, Cirebon, Indonesia; 5 Department of Physiology and Medical Biochemistry, Faculty of Medicine, Universitas Airlangga, Surabaya, Indonesia

**Keywords:** Immunotherapy, systematic review, biomarker, overall survival, progression-free survival, drug response

## Abstract

**Background and objective:**

Pembrolizumab has shown significant therapeutic benefit in advanced non-small cell lung cancer (NSCLC), but it remains uncertain which patients will benefit the most, and recent data suggest that programmed death-ligand 1 (PD-L1) expression as a single predictive biomarker is insufficient. This systematic review and meta-analysis looked at the safety and efficacy of pembrolizumab in PD-L1-positive advanced NSCLC patients, with a particular focus on disparities in treatment response to PD-L1 level of expression and demographic characteristics.

**Method:**

According to the PRISMA 2020 guidelines, six large databases were searched up to March 2025 for randomized controlled trials comparing pembrolizumab with chemotherapy in patients with such conditions. Overall survival (OS) and progression-free survival (PFS) were chosen as primary outcomes, and overall response rate (ORR) and safety profiles as secondary endpoints. A meta-analysis was conducted using a random-effects model, and the Cochrane risk of bias (ROB2) tool was employed to evaluate study quality. Seven randomized controlled trials involving 4,900 patients were included in the analysis.

**Key results:**

Pembrolizumab had a substantially better performance compared to chemotherapy for all the measures of efficacy: OS (hazard ratio (HR) 0.65, 95 % confidence interval (CI): 0.57 to 0.73, *P* < 0.00001), PFS (HR 0.55, 95 % CI: 0.42 to 0.72, *P* < 0.0001) and ORR (relative risk 2.10, 95 % CI: 1.51 to 2.93, *P* < 0.0001). Subgroup analysis showed greater survival benefit in patients younger than 65 years (OS HR 0.55) compared to patients aged 65 and older (OS HR 0.72), and in females (OS HR 0.44) compared to males (OS HR 0.67). Of most significant importance, those with PD-L1 expression <1 % also saw considerable benefit in survival (OS HR 0.60), casting doubts over the existing biomarker-based selection criteria.

**Conclusion:**

In conclusion, pembrolizumab achieves clinically meaningful survival benefits and an acceptable toxicity in PD-L1-positive advanced NSCLC. The high efficacy observed even in low PD-L1 expressers, and demographic differences in drug response, suggest that existing patient selection criteria could potentially be extended. These findings justify the application of a more advanced approach involving multiple biomarkers for more precise treatment allocation.

## Introduction

Lung cancer remains the leading cause of cancer-related deaths worldwide, with non-small cell lung cancer (NSCLC) accounting for roughly 85 % of all cases [1,2]. Over the past decade, treatment strategies for advanced NSCLC have shifted dramatically, moving from conventional chemotherapy toward personalized targeted therapies and immune checkpoint inhibitors (ICIs) [3-5]. These advances have translated into meaningful improvements in clinical outcomes, offering durable treatment responses and prolonged survival for patient groups that previously had very limited options [6,7].

Among immune checkpoint pathways, the programmed cell death protein-1 (PD-1) and its ligand PD-L1 have emerged as central therapeutic targets [8,9]. Their interaction functions as a key immune checkpoint, preventing excessive T-cell activation and maintaining immune balance [10]. However, tumour cells often exploit this mechanism by overexpressing PD-L1, thereby suppressing T-cell-mediated anti-tumour responses and escaping immune surveillance [11-13]. Blocking this pathway with monoclonal antibodies has proven effective in restoring immune-driven tumour destruction across multiple cancer types [14,15].

Pembrolizumab, a humanized IgG4-κ monoclonal antibody that targets PD-1, has demonstrated substantial clinical benefit in advanced NSCLC [16,17]. Results from the pivotal KEYNOTE trials consistently show improved Overall survival (OS) and progression-free survival (PFS) compared with standard chemotherapy, leading to its approval and widespread use in clinical practice [18-20]. These findings have established pembrolizumab as a cornerstone of first-line therapy for advanced NSCLC, fundamentally reshaping treatment guidelines and prognostic expectations [21,22].

Despite these advances, important challenges remain in refining patient selection for pembrolizumab. Currently, PD-L1 expression assessed by immunohistochemistry serves as the primary predictive biomarker, with tumour proportion scores (TPS) ≥50 % indicating the highest likelihood of response to monotherapy [23,24]. Yet, increasing evidence suggests that PD-L1 alone is insufficient for guiding treatment. Some patients with little or no PD-L1 expression achieve meaningful responses, while others with high expression levels derive limited benefit [25,26]. This paradox underscores the complexity of tumour-immune interactions, which cannot be fully explained by a single biomarker [27,28].

Clinical data also reveal wide variability in treatment outcomes across patient subgroups, indicating that demographic and molecular factors beyond PD-L1 play a role [29-31]. For instance, age and sex have been suggested as potential modifiers of immunotherapy efficacy, but these influences remain underexplored in systematic analyses [32-35]. In addition, long-term follow-up data from pivotal trials, along with emerging real-world evidence, are providing new insights into pembrolizumab’s clinical profile that call for updated evaluation [36-38].

Although several meta-analyses have assessed pembrolizumab in NSCLC, most included unselected patient populations or did not focus specifically on PD-L1-positive disease using the most contemporary data [39,40]. A critical knowledge gap therefore persists regarding the true therapeutic value of pembrolizumab in patients with PD-L1-positive advanced NSCLC, particularly in light of evolving biomarker limitations and patient-related factors influencing treatment response [41,42].

To address this gap, we conducted a systematic review and meta-analysis to evaluate the efficacy and safety of pembrolizumab in PD-L1-positive advanced NSCLC, with a particular focus on clinical and molecular predictors of response. Our primary objective was to synthesize evidence from randomized controlled trials to assess survival outcomes across different PD-L1 expression thresholds and patient subgroups. Secondary objectives included a comprehensive evaluation of safety and an examination of demographic factors related to treatment outcomes. Our analysis indicates that pembrolizumab significantly improves survival across all PD-L1 expression levels, with unexpected benefits in traditionally “biomarker-negative” patients and distinct response patterns linked to demographic characteristics. These findings challenge current patient selection paradigms and highlight the need for multidimensional biomarker strategies in the era of precision immunotherapy.

## Research design

A comprehensive literature search was conducted across multiple electronic databases, including PubMed/MEDLINE, Scopus, the Cochrane Library, Web of Science, ScienceDirect and EBSCO, covering publications from inception through March 2025. The search strategy combined Medical Subject Headings (MeSH) with free-text keywords such as “Non-Small Cell Lung Cancer”, “Pembrolizumab” *and* “PD-L1”, using Boolean operators (AND, OR) to ensure sensitivity and specificity. To minimize publication bias, additional gray literature sources were explored, including institutional repositories and relevant scientific platforms.

## Eligibility criteria

Studies were considered eligible if they met the following criteria: 1) Population: adults with PD-L1-positive NSCLC with histologic confirmation; 2) Intervention: pembrolizumab monotherapy or combination therapy in Phase II/III randomized controlled trials; 3) Control: chemotherapy; 4) Outcomes: OS, PFS, overall response rate (ORR), and safety outcomes; 5) Language: English-language publications.

Study selection was independently performed by two reviewers following a predefined protocol. Disagreements were resolved by discussion and, when necessary, consultation with a third reviewer.

## Data analysis

Data were extracted on study design, patient demographics, intervention details, and clinical outcomes. The methodological quality of included studies was assessed using the revised Cochrane Risk of Bias tool (RoB 2), which evaluates five domains of potential bias. Statistical analyses were performed using Review Manager (RevMan) version 5.4. Hazard ratios (HRs) with 95 % confidence intervals (CIs) were used for time-to-event outcomes, Overall survival (OS) and progression-free survival (PFS), while relative risks were used for dichotomous outcomes (ORR and safety). Heterogeneity was assessed using the *I*^2^ statistic, which guided the choice between fixed- and random-effects models. Prespecified subgroup analyses were conducted according to PD-L1 expression levels and key patient characteristics.

## Results and discussion

### Study selection and characteristics

**Figure 1.**
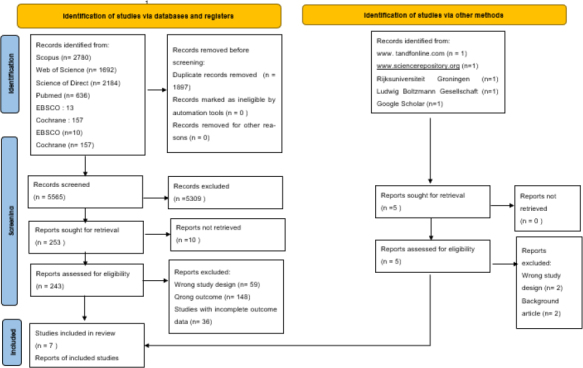
PRISMA flow diagram of literature screening and study selection

Preferred reporting items for systematic reviews and meta-analyses (PRISMA) flow diagram of literature screening and study selection is shown in Figure 1. The initial search identified 7,459 records, along with 5 additional records from other sources. After removing duplicates, 5,562 studies were screened, of which 7 randomized controlled trials (RCTs) published between 2016 and 2023 met the inclusion criteria, which are summarized in Table 1. In total, 4,900 patients were analysed, with 2,084 receiving pembrolizumab monotherapy and 1,816 assigned to chemotherapy as the control arm.

**Table 1.** Characteristics of included studies

Ref. Year	Trial ID KEYNOTE	Phase	Treatment arms	Total, *n*	Median follow-up, months	Sex, *n* / %		Histology, n /%		ECOG performance status, *n* / %		Smoking status, *n* / %		Geographic region, *n* / %		
														East Asia	Europe North America	Other
						Male	Female	Squamous	Non-squamous			Current/former	Never			
										0	1					
[43] 2016	010	III	Pembrolizumab 2 mg *vs.* 10 mg *vs.* Docetaxel	1,034	13.1	634 (61 %)	399 (39 %)	222 (21%)	724 (70 %)	348 (34 %)	678 (66 %)	833 (81 %)	190 (18 %)	190 (18%)	781 (76 %)	63 (6 %)
[44] 2016	021	II	Pembrolizumab + Chemotherapy *vs.* Chemotherapy	123	10.6	48 (39 %)	75 (61 %)	0 (0%)	121 (98 %)	53 (43 %)	69 (56 %)	99 (80 %)	24 (20 %)	0 (0%)	123 (100 %)	0 (0 %)
[22] 2016	024	III	Pembrolizumab *vs.* Chemotherapy	305	59.9	187 (61 %)	118 (39 %)	56 (18 %)	249 (82 %)	107 (35 %)	197 (65 %)	280 (92 %)	24 (8 %)	40 (13 %)	265 (87 %)	0 (0 %)
[45] 2019	042	III	Pembrolizumab *vs.* Chemotherapy	1,274	12.8	902 (71 %)	372 (29 %)	492 (39 %)	782 (61 %)	390 (31 %)	884 (69 %)	1,041 (82 %)	282 (22%)	370 (29 %)	286 (22 %)	618 (49 %)
[46] 2018	407	III	Pembrolizumab + Chemotherapy *vs.* Placebo + Chemotherapy	559	7.8	455 (81 %)	104 (19 %)	546 (98 %)	13 (2 %)	163 (29 %)	396 (71 %)	518 (93 %)	41 (7%)	106 (19 %)	453 (81 %)	0 (0 %)
[47] 2023	033	III	Pembrolizumab *vs.* Docetaxel	425	22.3	321 (76 %)	104 (24 %)	170 (40 %)	247 (58 %)	6 9 (16 %)	356 (84 %)	354 (83 %)	71 (17%)	358 (84 %)	67 (16 %)	0 (0 %)
[48] 2018	189	III	Pembrolizumab + Chemotherapy *vs*. Placebo + Chemotherapy	616	10.5	363 (59 %)	253 (4 1%)	0 (0 %)	606 (98 %)	266 (43 %)	346 (56 %)	543 (88 %)	73 (12%)	10 (2 %)	531 (86 %)	75 (12 %)

### Risk of bias assessment

**Figure 2.**
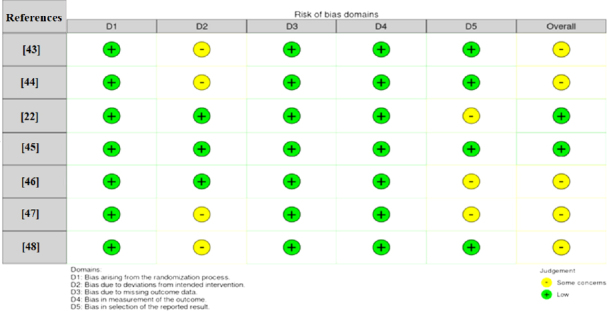
Assessment of risk of bias

Risk of bias assessment using the Cochrane ROB2 tool determined that one study showed low risk across all domains. The majority of studies revealed "some concerns," primarily in Domain 2 (bias resulting from deviations from the planned intervention) and Domain 5 (bias introduced through the study's rollout), as shown in Figure 2. This pattern aligns with findings reported in other systematic reviews of immunotherapy.

Domain 2 problems were due to the following causes: 1) Open-label study designs that would influence patient/clinician behaviour; 2) High crossover rates from control to intervention group (range: 20.3 to 66 %); 3) Differential discontinuation patterns between treatment groups.

Domain 5 concerns were related to protocol amendments, post-hoc subgroup analyses, and selective outcome reporting. These issues, although consistent with broader challenges in immunotherapy research, did not significantly undermine the overall quality of the evidence.

### Primary efficacy outcomes

#### Objective response rate

**Figure 3.**
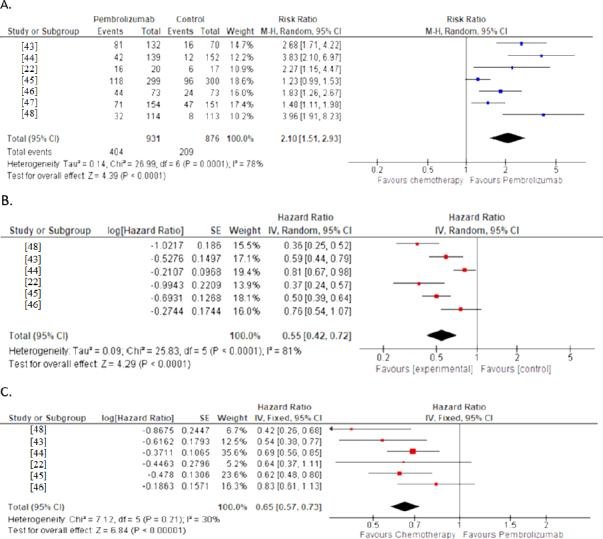
Forest plots illustrating survival outcomes in randomized clinical trials (RCTs): (A) ORR in RCTs,

All seven trials reported ORR for PD-L1-positive tumours. Pembrolizumab doubled the likelihood of objective response compared with chemotherapy (RR 2.10, 95 % CI 1.51-2.93; *P* < 0.0001), with 404 responses among 931 pembrolizumab patients versus 209 among 876 control patients (Figure 3A). Considerable heterogeneity (*I*^2^ = 78 %, *P* = 0.0001) likely reflected variation in PD-L1 thresholds and patient populations across studies.

#### Total survival

Six studies provided OS data for the PD-L1-positive group. Pembrolizumab monotherapy achieved a clinically important 35 % reduction in hazard of death compared with chemotherapy (HR 0.65, 95 % CI: 0.57-0.73, *P* < 0.00001) as observed in Figure 3B. The analysis showed low to moderate heterogeneity (*I*^2^ = 30 %, *P* = 0.21), favouring homogeneous survival benefits across study populations. This is a notable clinical benefit with robust statistical significance.

#### Progression-free survival

PFS analysis of six studies demonstrated that pembrolizumab significantly reduced the risk of disease progression or death by 45 % (HR 0.55, 95 % CI: 0.42-0.72, *P* < 0.0001) as illustrated in Figure 3C. However, high heterogeneity was observed (*I*^2^ = 81 %, *P* < 0.00001), suggesting variability in progression patterns across different patient populations and study designs. Despite this heterogeneity, the substantial effect size and statistical significance support the superior efficacy of pembrolizumab in delaying disease progression.

### Secondary outcomes

#### Safety analysis

In the combined safety analysis, 4,168 patients in the pembrolizumab arm and 3,632 patients in the control arm were included in seven randomized trials. Most patients in both arms experienced adverse events of any grade, with pembrolizumab having an evident but manageable safety profile compared to chemotherapy controls, as summarized in Table 2.

**Table 2.** Overall safety summary and treatment-related adverse events

Ref.	Treatment arms	Total, *n*	Any grade AEs, *n* / %	Grade 3-5 AEs, *n* / %	Treatment-related deaths, *n* / %	Discontinuation due to AEs, n / %
[43]	Pembrolizumab 2 mg	344	215/339 (63.4 %)	43/339 (12.7 %)	3/344 (0.9 %)	15/344 (4.4 %)
	Docetaxel	343	251/309 (81.2 %)	109/309 (35.3 %)	5/343 (1.5 %)	31/343 (9.0 %)
[44]	Pembrolizumab + Chemo	60	55/59 (93.2 %)	23/59 (39.0 %)	1/60 (1.7 %)	6/60 (10.0 %)
	Chemotherapy	63	56/62 (90.3 %)	16/62 (25.8 %)	2/63 (3.2 %)	8/63 (12.7 %)
[22]	Pembrolizumab	154	118/154 (76.6 %)	48/154 (31.2 %)	2/154 (1.3 %)	21/154 (13.6 %)
	Chemotherapy	151	135/150 (90.0 %)	80/150 (53.3 %)	3/151 (2.0 %)	16/151 (10.6 %)
[45]	Pembrolizumab	637	399/636 (62.7 %)	113/636 (17.8 %)	13/637 (2.0 %)	57/637 (8.9 %)
	Chemotherapy	637	553/615 (89.9 %)	252/615 (41.0 %)	14/637 (2.2 %)	58/637 (9.1 %)
[46]	Pembrolizumab + Chemo	278	273/278 (98.2 %)	194/278 (69.8 %)	10/278 (3.6 %)	37/278 (13.3 %)
	Placebo + Chemo	281	274/280 (97.9 %)	191/280 (68.2 %)	6/281 (2.1 %)	18/281 (6.4 %)
[47]	Pembrolizumab	213	149/213 (70.0 %)	24/213 (11.3 %)	4/213 (1.9 %)	21/213 (9.9 %)
	Docetaxel	212	174/198 (87.9 %)	94/198 (47.5 %)	4/212 (1.9 %)	15/212 (7.1 %)
[48]	Pembrolizumab + Chemo	410	404/405 (99.8 %)	272/405 (67.2 %)	27/410 (6.6 %)	NR
	Placebo + Chemo	206	200/202 (99.0 %)	133/202 (65.8 %)	27/206 (13.1 %)	NR

#### Overall safety profile

Grade 3-5 adverse events had variable incidence between trials, with severe toxicity of 11.3 to 67.2 % in patients treated with pembrolizumab, compared with 25.8 to 68.2 % in control arms, as summarized in Table 2. Treatment discontinuation due to adverse events was observed in pembrolizumab-treated patients at frequencies ranging from 4.4% to 13.6%, compared with frequencies ranging from 6.4 to 12.7 % in the control arms. Treatment-related mortality remained infrequent in both groups:

- Pembrolizumab: 0.9 to 6.6 % across trials,
- Control: 1.5 to 13.1 %,
- No overall mortality difference by treatment groups with pooled analysis.

### Immune-related adverse events

Immune-mediated adverse events of any grade occurred more frequently in pembrolizumab-treated patients compared to controls, with incidence rates ranging from 20.1 to 34.4 **%** versus 5.3 to 11.3 **%** in control groups. Grade 3-5 immune-related adverse events showed rates of 5.1 to 13.6 **%** in pembrolizumab patients compared to 0.7 to 3.2 **%** in controls.

Pneumonitis emerged as the most clinically significant immune-related adverse event (see Table 3): any grade: 4.0 to 9.9 **%** in pembrolizumab patients versus 0 to 2.5 **%** in controls; Grade 3-5: 1.7 to 3.2 **%** versus 0 to 2.0 **%** in control groups. Endocrine toxicities demonstrated characteristic patterns: Hypothyroidism: 7.9 to 15.3 **%** in pembrolizumab versus 0.3 to 11.9 **%** in controls; Grade 3-5 hypothyroidism: 0 to 1.0 **%** versus 0 to 0.5 **%** in controls; Hyperthyroidism: 4.0 to 8.0 **%** versus 0 to 3.0 **%** in controls.

**Table 3.** Immune-mediated adverse events

Ref.	Treatment arms	Total, *n*	Any grade irAEs, *n* / %	Grade 3-5 irAEs, n / %	Most common irAEs (≥5 % incidence)
[43]	Pembrolizumab, 2 mg	344	69/344 (20.1 %)	NR	Hypothyroidism (8 %), hyperthyroidism (4 %), pneumonitis (4 %)
	Docetaxel	343	NR	NR	Minimal immune-related events
[44]	Pembrolizumab + Chemo	60	13/59 (22.0 %)	3/59 (5.1 %)	Hypothyroidism (15 %), hyperthyroidism (8 %), pneumonitis (5 %)
	Chemotherapy	63	7/62 (11.3 %)	0/62 (0 %)	Minimal immune-related events
[22]	Pembrolizumab	154	53/154 (34.4 %)	21/154 (13.6 %)	Hypothyroidism (10.4 %), hyperthyro-idism (7.1 %), pneumonitis (8.4 %)
	Chemotherapy	151	8/150 (5.3 %)	1/150 (0.7 %)	Minimal immune-related events
[45]	Pembrolizumab	637	177/636 (27.8 %)	51/636 (8.0 %)	Pneumonitis (22 patients), severe skin reactions (11 patients), hepatitis (7 patients)
	Chemotherapy	637	44/615 (7.2 %)	9/615 (1.5 %)	Minimal immune-related events
[46]	Pembrolizumab + Chemo	278	80/278 (28.8 %)	30/278 (10.8 %)	Hypothyroidism (7.9 %), pneumonitis (6.5 %), colitis (2.5 %)
	Placebo + Chemo	281	24/280 (8.6 %)	9/280 (3.2 %)	Minimal immune-related events
[47]	Pembrolizumab	213	61/213 (28.6 %)	13/213 (6.1 %)	Hypothyroidism (13.1 %), pneumonitis (9.9 %), hepatitis (1.9 %)
	Docetaxel	212	12/198 (6.1 %)	3/198 (1.5 %)	Minimal immune-related events
[48]	Pembrolizumab + Chemo	410	NR	NR	NR
	Placebo + Chemo	206	NR	NR	NR

### Specific adverse event profiles

Hematologic toxicities revealed significant differences favouring pembrolizumab: Neutropenia occurred substantially less frequently in pembrolizumab patients (0 to 38 %) compared to chemotherapy controls (6 to 24 %); Grade 3-5 neutropenia showed even more pronounced differences (pembrolizumab: 0 to 23 %; control: 2 to 25 %); Similar patterns were observed for anemia and other hematologic parameters.

Gastrointestinal toxicities showed divergent patterns: Nausea occurred with variable frequency in pembrolizumab patients (1 to 56 %) compared to chemotherapy controls (13 to 52 %); Diarrhea demonstrated variable incidence (pembrolizumab: 2 to 31 %; control: 7 to 23 %); Immune-mediated colitis occurred in 0.6 to 3.9 % of pembrolizumab patients compared to 0 % to 1.4 % in controls. The safety analysis confirmed pembrolizumab's distinct toxicity profile, characterized by immune-related adverse events while demonstrating reduced incidence of traditional chemotherapy-related toxicities. This differential toxicity profile supports the clinical utility of pembrolizumab, particularly in patients who may not tolerate intensive chemotherapy regimens.

### Subgroup analyses

#### Age-stratified analysis

Age had a consistent and significant impact on pembrolizumab effectiveness in both survival endpoints, as analysed by PFS and OS, as demonstrated by Tables 4 and 5, respectively. In OS, patients younger than 65 years received significantly more benefit (HR 0.55; 95 % CI 0.46 to 0.65; *P*<0.00001) compared to patients 65 years or older (HR 0.72; 95 % CI 0.59 to 0.88; *P* = 0.001). This age-related trend was also evident in the PFS analysis, with the younger group showing a significantly greater benefit (HR 0.46; 95 % CI 0.38-0.57; *P* <0.00001) compared to older patients (HR 0.68; 95 % CI 0.55 to 0.85; *P* = 0.0005).

**Table 4.** Analysis of OS in patient subgroups with varying clinical characteristics

Population	Subgroup	No. of studies	HR	95 % CI	*I*^2^ / %	*p* value
Age-Stratified Analysis						
Age < 65 years	Total	3	0.55	0.46-0.65	44	<0.00001
Age ≥ 65 years	Total	3	0.72	0.59-0.88	0	0.001
Sex-Based Analysis						
Male	Total	3	0.67	0.57-0.79	0	<0.00001
Female (PD-L1 ≥50 %)	Total	3	0.44	0.24-0.82	87	0.009
Performance Status Analysis						
ECOG PS 0	Total	3	0.60	0.47-0.77	38	<0.0001
ECOG PS 1	Total	3	0.66	0.57-0.77	0	<0.00001
PD-L1 Expression Analysis						
PD-L1 TPS < 1 %	Total	2	0.60	0.43-0.83	0	0.002
PD-L1 TPS ≥ 1 %	Total	5	0.71	0.60-0.84	59	<0.0001
PD-L1 TPS 1-49 %	Total	2	0.56	0.40-0.78	0	0.0006

**Table 5.** Analysis of PFS in patient subgroups with varying clinical characteristics

Population	Subgroup	No. of studies	HR	95 % CI	*I*^2^ / %	*p* value
Age-stratified analysis						
Age < 65 years	Total	2	0.46	0.38-0.57	0	<0.00001
Age ≥ 65 years	Total	2	0.68	0.55-0.85	0	0.0005
Sex-based analysis						
Male	Total	2	0.61	0.51-0.73	0	<0.00001
Female	Total	2	0.43	0.32-0.56	0	<0.00001
Performance status analysis						
ECOG PS 0	Total	2	0.47	0.36-0.62	0	<0.00001
ECOG PS 1	Total	2	0.59	0.49-0.70	0	<0.00001
PD-L1 expression analysis						
PD-L1 TPS < 1 %	Total	2	0.60	0.43-0.83	%	0.002
PD-L1 TPS ≥ 1 %	Total	5	0.74	0.54-1.01	90	0.05
PD-L1 TPS 1-49 %	Total	2	0.56	0.43-0.73	0	<0.0001

Heterogeneity was low for the young population for OS (*I*^2^=44 %) and zero for PFS (*I^2^*=0 %), while older patients demonstrated no heterogeneity across studies (*I^2^*=0 % for both endpoints), supporting strong and consistent age-related treatment effects.

#### Sex-based analysis

Gender stratification revealed dramatic differences in pembrolizumab efficacy magnitude among endpoints. In the OS of the PD-L1 ≥50 % subgroup, female patients exhibited exceptional benefit (HR 0.44; 95 % CI 0.24-0.82; *P*=0.009), significantly better than that of male patients (HR 0.67; 95 % CI 0.57-0.79; *P*<0.00001). The female *OS* analysis revealed, nonetheless, significant heterogeneity (*I^2^*=87 %), reflecting heterogeneity of response to treatment across studies.

This sex difference occurred uniformly in PFS, where females fared better (HR 0.43; 95 % CI 0.32-0.56; *P*<0.00001) than males (HR 0.61; 95 % CI 0.51-0.73; *P*<0.00001) (Tables 2 and 3). Notably, the PFS analysis did not reveal any heterogeneity within either sex stratum (I^2^=0 %), indicating consistent treatment effects within each gender group.

#### Performance status analysis

Eastern Cooperative Oncology Group (ECOG) performance status indicated pembrolizumab efficacy across all functional status levels with relatively small variation between groups. OS analysis indicated significant benefit in both ECOG 0 (HR 0.60; 95 % CI 0.47 to 0.77; *P*<0.0001) and ECOG 1 patients (HR 0.66; 95 % CI 0.57-0.77; *P* <0.00001). The ECOG 0 subgroup demonstrated moderate heterogeneity (*I*^2^ = 38 %), while ECOG 1 patients demonstrated total homogeneity (*I*^2^ = 0 %).

PFS also concorded with results in ECOG 0 patients HR 0.47 (95 % CI 0.36 to 0.62; *P* <0.00001) and ECOG 1 patients HR 0.59 (95 % CI 0.49 to 0.70; *P* <0.00001) (Tables 2 and 3). Notably, both PFS subgroups did not have heterogeneity (*I^2^* = 0 %), implying extremely consistent treatment effects across the varying baseline functional status.

### PD-L1 expression analysis

The stratification of tumour score proportion has demonstrated the efficacy of pembrolizumab across the full spectrum of PD-L1 levels, revealing unexpected trends that contradict traditional biomarker-driven selection frameworks. OS and PFS analyses in Tables 4 and 5, respectively, further confirmed that patients with a TPS of less than 1 % experienced significant benefits, indicated by a hazard ratio of 0.60 (95 % CI 0.43 to 0.83; *P* = 0.002). In contrast, those with TPS ranging from 1 to 49 % exhibited similar efficacy, with a hazard ratio of 0.56 (95 % CI 0.40 to 0.78; P=0.0006).

Patients classified with TPS of 1 % or greater showed a significant, albeit less pronounced, benefit, reflected by a hazard ratio of 0.71 (95 % CI 0.60 to 0.84; *P* <0.0001), accompanied by moderate heterogeneity (*I*^2^=59 %). In the cohort of patients with TPS less than 1 %, the analysis of progression-free survival indicated a consistent benefit, with a hazard ratio of 0.60 (95 % CI 0.43 to 0.83; *P* = 0.002). Conversely, patients with TPS between 1 and 49 % demonstrated comparable efficacy, with a hazard ratio of 0.56 (95 % CI 0.43-0.73; *P* <0.0001).

The subgroup of patients with TPS of 1 % or more exhibited only marginal statistical significance, as evidenced by a hazard ratio of 0.74 (95 % CI 0.54 to 1.01; *P* = 0.05), alongside a notably high level of heterogeneity (*I*^2^=90 %). This surprising finding raises questions about established PD-L1-based selection criteria and highlights the need for further exploration of alternative biomarker approaches. The consistent benefit observed across various PD-L1 expression levels, particularly within the historically regarded "biomarker-negative" group (TPS <1 %), advocates for a broader consideration of therapeutic options beyond the existing PD-L1-centric selection paradigms.

### Sensitivity analysis and publication bias

Sensitivity analyses were conducted by systematically removing each study individually, which revealed that the primary outcomes remained consistent throughout these exclusions. This consistency demonstrates minimal sensitivity and reinforces the reliability and stability of the findings. The prognostic factors in the overall study population remained unaffected by these sensitivity analyses.

**Figure 4.**
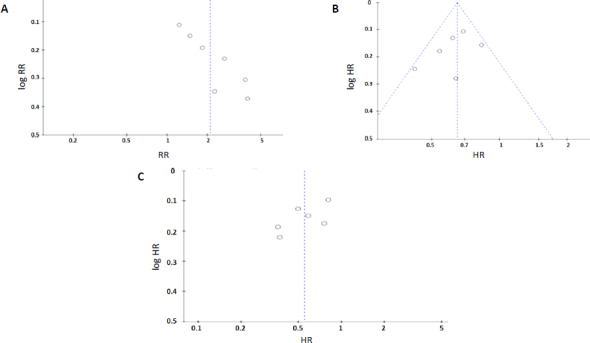
Funnel plots for (A) ORR (B) OS (C) PFS between pembrolizumab and chemotherapy

Assessment of publication bias showed no substantial bias, as indicated by the symmetrical funnel plots for both OS and PFS (Figure 4), as well as for the subgroup analyses of these endpoints.

These results strengthen the validity of the meta-analysis conclusions and suggest that the treatment effects observed are robust and not substantially influenced by selective publication practices.

## Discussion

### Primary findings in context of previous research

A pooled analysis revealed a 45 % reduction in the risk of disease progression with pembrolizumab (HR 0.55, 95 % CI: 0.42-0.72). This effect size was not only consistent with but, in some cases, stronger than results from individual trials such as KEYNOTE-024 (HR 0.50, 95 % CI: 0.37 to 0.68) and KEYNOTE-042 (HR 0.81, 95 % CI: 0.71 to 0.93) [46]. The twofold increase in objective response rate (RR 2.10, 95 % CI: 1.51 to 2.93) further illustrates pembrolizumab’s potent antitumor activity, aligning with the established mechanism of checkpoint blockade that restores T-cell-mediated immune surveillance [49].

One of the most unexpected findings was that PD-L1 expression did not consistently predict treatment response. Patients with a TPS below 1%, typically considered “biomarker-negative,” derived a clear survival benefit (OS HR 0.60, 95% CI: 0.43 to 0.83), in some instances comparable to those with higher PD-L1 expression [50]. This challenges the prevailing paradigm established by early pembrolizumab trials, which restricted monotherapy to PD-L1-high populations (TPS ≥50 %) and still guides FDA approvals [51]. Our results support accumulating real-world evidence showing that PD-L1 alone cannot capture the complexity of tumour-immune interactions that shape responses to immunotherapy [52].

Age and sex also emerged as significant modifiers of treatment effect. Younger patients (<65 years) experienced substantially greater benefit than older patients (OS HR 0.55 *vs.* 0.72), suggesting immune senescence may blunt checkpoint inhibitor efficacy. This aligns with evidence of reduced T-cell function and immune surveillance with aging [53]. Similarly, women demonstrated greater survival benefit (OS HR 0.44 *vs.* 0.67 in men), consistent with the known sexual dimorphism in immune responses [54]. These findings suggest that demographic factors, often overlooked in patient selection, may meaningfully shape outcomes and should be considered alongside molecular biomarkers.

The most clinically significant implication of our study is the strong efficacy signal in patients with low or negative PD-L1 expression, a population currently excluded from pembrolizumab monotherapy under most treatment guidelines. Extending eligibility to this group could expand therapeutic options for up to 30 to 40 % of advanced NSCLC patients who are currently treated with chemotherapy alone [55].

The biological basis of this PD-L1 paradox is likely multifactorial, involving intratumoral heterogeneity, temporal variation in PD-L1 expression, and inflammation-induced changes during therapy [56]. Other contributors may include immune cell PD-L1 expression and additional checkpoint pathways [57]. Together, these factors highlight the limitations of PD-L1 as a standalone biomarker.

Our subgroup analyses provide biologically plausible insights that have immediate clinical relevance. Younger patients’ stronger immune systems likely underpin their improved responses [58], whereas immune senescence in older individuals - characterized by thymic atrophy, T-cell exhaustion, and chronic inflamemation - may diminish benefit [59]. Likewise, women’s more active immune responses, while predisposing them to autoimmunity, may increase sensitivity to checkpoint blockade [60]. These differences emphasize the need for treatment strategies that account for patient demographics as well as tumour biology.

Safety findings were consistent with prior reports. Pembrolizumab was associated with immune-related toxicities such as pneumonitis (4 to 9.9 %) and hypothyroidism (7.9 to 15.3 %), but these events were generally manageable. Importantly, pembrolizumab carried a markedly lower risk of hematologic toxicities such as severe neutropenia (0 to 23 % *vs.* 2 to 25 % with chemotherapy) [61]. This favourable safety profile is particularly relevant for older patients, those with comorbidities, or individuals with poor performance status who may not tolerate chemotherapy.

The high heterogeneity observed in PFS analysis reflects differences in trial populations, PD-L1 assays, and comparator regimens. While this reduces precision, it increases generalizability by better reflecting real-world variation [62]. Still, reliance on trial populations limits applicability to routine practice, where patients often have greater comorbidity and lower performance status. The geographic underrepresentation of Asian populations further limits the external validity [63].

Our findings underscore the urgent need for more sophisticated biomarkers beyond PD-L1. Composite approaches incorporating tumour mutational burden, immune gene signatures, circulating biomarkers, and AI-driven imaging hold promise [64]. Advances in liquid biopsy technology, including circulating tumour DNA and immune profiling, could enable real-time monitoring of treatment response [65].

Although our analysis focused on pembrolizumab monotherapy, future progress will likely depend on rational combination strategies with chemotherapy, anti-angiogenic agents, and novel immunomodulators [66]. The observed demographic differences suggest tailoring these combinations to patient subgroups may maximize benefit [67]. Integrating real-world evidence, registry data, and pragmatic trials will be crucial to understanding long-term outcomes and optimizing sequencing in diverse patient populations [68].

Taken together, our results signal a paradigm shift away from reliance on a single biomarker toward multifactorial patient selection models. Incorporating demographic, clinical, and molecular variables into integrated risk prediction frameworks could enhance precision in treatment decisions [69]. Machine learning and AI offer powerful tools to synthesize these diverse data sources and may reveal novel signatures of response that are not captured by traditional analyses [70].

From a regulatory and clinical practice perspective, the clear benefits observed in PD-L1-low patients raise the possibility of broadening pembrolizumab monotherapy indications. Such a shift will require balancing clinical efficacy with cost, health system capacity, and equity of access. Collaborative efforts among regulators, professional societies, and healthcare systems will be crucial to translating these insights into enhanced patient care.

Finally, our study has limitations. Reliance on trial-level rather than individual patient data constrained our ability to explore confounding factors and limited biomarker analyses, such as tumour mutational burden, which was inconsistently reported. These gaps highlight the importance of ongoing research to refine patient selection and therapeutic strategies.

## Conclusions

This meta-analysis provides robust evidence that pembrolizumab significantly improves overall survival, progression-free survival, and objective response rates in patients with advanced non-small cell lung cancer exhibiting PD-L1-positive expression. The treatment exhibits a tolerable safety profile, primarily characterized by immune-related adverse events rather than the typical cytotoxic side effects. Notably, the subgroup analyses presented here suggest that pembrolizumab offers considerable clinical benefits not only to patients with high PD-L1 expression but also to those with low or even undetectable PD-L1 levels—this finding challenges the established paradigms of biomarker-driven treatment. The variability in treatment responses among different age and gender cohorts highlights the biological diversity in patient reactions to immunotherapy and reinforces the need for developing more personalized therapeutic strategies. These findings underscore the limitations of PD-L1 expression as a solitary predictive biomarker and stress the importance of incorporating additional testing methods, such as tumour mutational burden, immune gene profiling, and liquid biopsy technologies into clinical practice. Overall, while pembrolizumab remains a cornerstone treatment for advanced NSCLC, optimizing patient selection through multidimensional biomarker strategies will be crucial to enhance therapeutic outcomes and accelerate personalized cancer treatment in the immunotherapy era.
